# Mutation Types and Aging Differently Affect Revertant Fiber Expansion in Dystrophic *Mdx* and *Mdx52* Mice

**DOI:** 10.1371/journal.pone.0069194

**Published:** 2013-07-24

**Authors:** Yusuke Echigoya, Joshua Lee, Merryl Rodrigues, Tetsuya Nagata, Jun Tanihata, Ashkan Nozohourmehrabad, Dharminder Panesar, Bailey Miskew, Yoshitsugu Aoki, Toshifumi Yokota

**Affiliations:** 1 Department of Medical Genetics, University of Alberta Faculty of Medicine and Dentistry, Edmonton, Alberta, Canada; 2 Department of Molecular Therapy, National Institute of Neuroscience, National Center of Neurology and Psychiatry, Kodaira, Tokyo, Japan; 3 Department of Physiology, Anatomy and Genetics, University of Oxford, Oxford, United Kingdom; 4 The Friends of Garrett Cumming Research and Muscular Dystrophy Canada HM Toupin Neurological Science Research Chair, Edmonton, Alberta, Canada; The Hospital for Sick Children, Canada

## Abstract

Duchenne muscular dystrophy (DMD), one of the most common and lethal genetic disorders, and the *mdx* mouse myopathies are caused by a lack of dystrophin protein. These dystrophic muscles contain sporadic clusters of dystrophin-expressing revertant fibers (RFs), as detected by immunohistochemistry. RFs are known to arise from muscle precursor cells with spontaneous exon skipping (alternative splicing) and clonally expand in size with increasing age through the process of muscle degeneration/regeneration. The expansion of revertant clusters is thought to represent the cumulative history of muscle regeneration and proliferation of such precursor cells. However, the precise mechanisms by which RFs arise and expand are poorly understood. Here, to test the effects of mutation types and aging on RF expansion and muscle regeneration, we examined the number of RFs in *mdx* mice (containing a nonsense mutation in exon 23) and *mdx52* mice (containing deletion mutation of exon 52) with the same C57BL/6 background at 2, 6, 12, and 18****months of age. *Mdx* mice displayed a significantly higher number of RFs compared to *mdx52* mice in all age groups, suggesting that revertant fiber expansion largely depends on the type of mutation and/or location in the gene. A significant increase in the expression and clustering levels of RFs was found beginning at 6****months of age in *mdx* mice compared with *mdx52* mice. In contrast to the significant expansion of RFs with increasing age, the number of centrally nucleated fibers and embryonic myosin heavy chain-positive fibers (indicative of cumulative and current muscle regeneration, respectively) decreased with age in both mouse strains. These results suggest that mutation types and aging differently affect revertant fiber expansion in *mdx* and *mdx52* mice.

## Introduction

Duchenne muscular dystrophy (DMD) is the most common genetic muscular disease and is characterized by progressive muscle degeneration. It occurs with a frequency of about 1 out of 3,500 boys, and is caused by mutations in the *dystrophin* (*DMD*) gene [1]–[3]. The mutation leads to progressive myopathy and muscle weakness coupled with cycles of muscle degeneration. Death eventually occurs due to severe respiratory and/or cardiac failure at approximately 20–30****years of age [4], [5]. There is currently no effective cure for DMD [6]–[8]. The *DMD* gene is located on the X-chromosome and is characterized as one of the largest and more complex genes in humans, containing 79 exons and spanning more than 2.4****million base pairs [9], [10]. The *DMD* gene encodes at least 18 protein isoform products of dystrophin with tissue specific alternative promoters [11]–[26]. The main skeletal muscle isoform is the largest known, consisting of 3,685-amino acids (427****kDa). In skeletal muscles, dystrophin plays a central role in organizing a multi-protein complex at the sarcolemma, and linking cytoskeleton proteins to extracellular matrix proteins [27], [28]. The full-length dystrophin protein can be divided into actin-binding NH_2_- (N) terminal, rod, cysteine-rich, and COOH- (C) terminal domains [14], [20]. Interestingly, most of the known functions of the protein are assigned to the N- and C- terminals and the cysteine-rich domain [29]. In contrast, the central rod domain, consisting of 24 spectrin repeats with 4 hinges, and spanning about half the length of the protein, appears to be less essential to proper function [30].

Most DMD mutations, which cause disruption of the open reading frame, occur within the central rod domain, thus preventing translation of the crucial C-terminal domain [31]–[33]. In the case of Becker muscular dystrophy (BMD), a milder form of muscular dystrophy also caused by mutations in the *DMD* gene, most mutations occur in the same rod domain regions, but the mutated mRNA transcripts preserve the open reading-frame and are thus translated into a truncated yet partially functional protein [34]. The *mdx* mouse, an animal model of human DMD, harbors a natural nonsense point mutation in exon 23, while the *mdx52* mouse has a deletion mutation of exon 52 owing to gene-targeting method [35]–[38]. *Mdx* mice were originally discovered with C57BL/10 background, but they were later backcrossed with C57BL/6 background for comparison with other mouse models including *mdx52* mice with C57BL/6 background [39]. Both mutants exhibit a lack of dystrophin expression and cycles of muscle degeneration/regeneration [40], [41]. Thus, the comparison between *mdx* and *mdx52* mice with the same genetic background should be quite useful in understanding pathogenic mechanisms dependent on different types of nonsense and exon-deletion mutations, which are found in approximately 10–15% and 50–60% of the DMD patients, respectively [42], [43].

Interestingly, skeletal muscles in most DMD patients and animal models including mouse and dog models display sporadic dystrophin-positive muscle fibers called “revertant fibers” (RFs) in an otherwise dystrophin-negative background [44]–[51]. Danko *et al* report distinct frequencies of RFs in different *mdx* mutants (*mdx2cv*, *3cv*, *4cv*, and *5cv* mutants) [50]. These dystrophin-positive fibers arise from spontaneous exon skipping (alternative splicing) with a loss of up to 30 exons, leading to the production of in-frame truncated proteins [52]. In *mdx* mice, these RFs expand with age through cycles of muscle degeneration/regeneration and the activation of muscle precursor cells [40], [52]. In human DMD patients, the increase of RFs correlates significantly with their age up to early teens [53]. These revertant events are thought to arise within a subset of muscle precursor cells which proliferate in response to muscle degeneration and participate in regeneration of muscle fibers [40]. These fibers often produce clonal clusters that can expand to up to 100 fibers measuring more than 1****mm in length by 18****months of age [52]. To date, there is no report of either genomic deletion or secondary mutation as the mechanism facilitating the restoration of dystrophin in RFs. Our previous studies using various transgenic mouse models, including micro-dystrophin transgenic *mdx* mice and utrophin overexpressing mice, as well as irradiated *mdx* models, clearly demonstrated that expansion of RF clusters is dependent on muscle regeneration [40], [54]–[57]. The expansion of RF clusters reflects the cumulative history of skeletal muscle regeneration and is thought to provide a useful index for functional evaluation of therapies that diminish muscle degeneration [40].

In this study, we employed two newly developed mouse models, *mdx* mice with C57BL/6 background, and *mdx52* mice [37], [39]. We compared their RF generation and long-term expansion up to 18****months of age. Interestingly, *mdx* mice exhibited a higher number of RFs compared to *mdx52* mice, indicating the occurrence of revertant events largely depends on the type of mutation present in the *DMD* gene. To our surprise, although both mouse models showed an increase in RFs through 18****months, the number of centrally nucleated fibers (CNFs) and embryonic myosin heavy chain (eMHC)-positive fibers (as cumulative and current indicators of muscle regeneration, respectively) decreased with age in both the strains except for an increase in CNFs between 2 to 6****months of age in *mdx* mice. Overall, the dynamics of muscle regeneration associated with age was more markedly altered in *mdx* than *mdx52* mice. These data reported here show that mutation types and aging differently affect RF expansion and muscle regeneration in *mdx* and *mdx52* mice.

## Materials and Methods

### Ethics Statement

All animal works were conducted according to relevant national and international guidelines. Animal study was approved by the Ethics Committee for the Treatment of Laboratory Animals of the National Center of Neurology and Psychiatry, and the Animal Care and Use Committee (ACUC) of the University of Alberta. All animals were euthanized by cervical dislocation by trained personnel.

### Animals

*Mdx* mice with C57BL/6 background, *mdx52* mice, and C57BL/6 mice as controls were used in this study [37], [39], [58]. The genetic background of *mdx* mice was changed into C57BL/6 as previously described [39]. Male and female *mdx* and *mdx52* mice at 2, 6, 12, and 18****months of age were used in this study. Homozygous mutation of the *DMD* gene was confirmed in female mice by genotyping. Male C57BL/6 mice at 2****months of age were used as controls. Mice were euthanized by cervical dislocation; then, tibialis anterior (TA) and gastrocnemius (GC) muscles were excised and immediately frozen in liquid nitrogen-cooled isopentane. Samples were stored at −80°C until used for immunohistochemistry and histochemistry.

#### Immunohistochemical analysisAntibodies

The rabbit polyclonal antibody against C-terminal domain (position at 3,661–3,677 amino acids) in human dystrophin was used to detect revertant fibers (Abcam, Bristol, UK). eMHC expressed in newly regenerated muscle fibers was detected with mouse monoclonal anti-rabbit developmental MHC antibody (Leica Biosystems, Newcastle upon Tyne, UK).

#### Immunohistochemistry

Dystrophin-positive RFs were detected by immunohistochemistry. Transverse frozen sections (7****μm thick) from TA and GC muscles were cut using a Leica CM1900 cryostat (Leica Micro-systems, Wetzlar, Germany). Serial sections were picked up on poly-L-lysine-coated glass microscope slides and air-dried for 30****min. Unfixed sections were then blocked in phosphate-buffered saline (PBS) with 20% goat serum, 0.1% TritonX-100 for one hour at room temperature. Dystrophin was detected with rabbit polyclonal primary antibody against human dystrophin C-terminal (1∶400) in the blocking solution by overnight incubation at 4°C. After washing 3 times with PBS, the primary antibody was detected with AlexaFluor^TM^ 488-conjugated goat anti-rabbit IgG secondary antibody (1∶2,000) (Molecular Probes, OR, USA) with one-hour room temperature incubation. Nuclear counterstaining was performed with 4',6-diamidino-2-phenylindole (DAPI) in a mounting agent (Vectashield; Vector Laboratories, CA, USA). Expression of eMHC and its co-localization with RFs were assessed by triple staining using anti-developmental MHC antibody (1∶20), anti-dystrophin antibody (1∶400), and DAPI after blocking with Mouse on Mouse (M.O.M.) blocking reagent (Vector Laboratories).

#### Assessment of RFs and eMHC-positive fibers

The muscle RF assessment was performed according to our previous study [40]. RFs and eMHC-positive fibers in entire TA or GC muscle sections were observed using a fluorescence microscope (Nikon Eclipse TE 2000-U; Nikon, Tokyo, Japan) with a 20X objective lens. Muscle fibers were regarded as dystrophin-positive only when more than half the membrane circumference was stained in cross-sections. RFs immediately adjacent to each other were characterized as a single cluster. For closer comparison of RFs and eMHC-positive fibers in mice of different groups, at least 8 serial sections at every 100****μm from the muscle belly were stained with antibodies to assess the following: the number of RFs in a cross-section, the number of revertant clusters, the maximum number of RFs in a cluster, and the number of eMHC-positive fibers. For data per mouse, the highest number among serial sections was averaged between left and right muscles for each of the aforementioned criteria. Large clusters of eMHC-positive fibers (clusters composed of more than one hundred eMHC-positive fibers, which arose due to severe degeneration and were independent of age) were excluded from counting.

### Hematoxylin and Eosin staining

Hematoxylin and eosin (HE) staining was performed with Mayer's hematoxylin and eosin solutions as previously described [41]. A DMR microscope (Leica Micro-systems) was used for bright field microscopy with a 20x objective lens. The percentage of CNFs was calculated in 300 to 1,000 fibers in each left and right muscle, and was averaged between two muscles per mouse.

### Statistical analysis

All data were reported as mean values ± standard deviation (S.D.). The statistical differences between the age groups or the strains were assessed by one-way ANOVA with a Tukey–Kramer multiple comparison test. Pearson correlation coefficient was performed between the number of RFs in a section, the number of RF clusters, and the maximum number of RFs in a single cluster. *P*<0.05 was considered statistically significant.

## Results

### Distinct patterns of revertant fiber expression and clustering in *mdx* and *mdx52* mice

**Figure 1:**
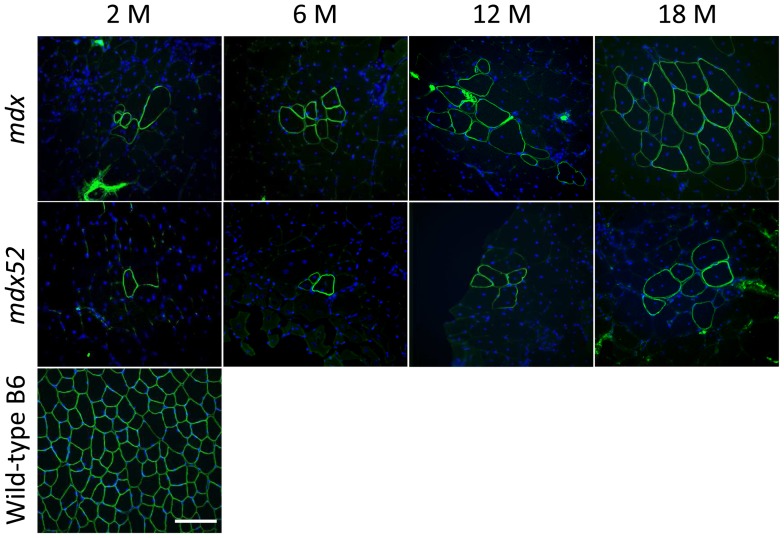
Dystrophin-positive revertant fibers with central nuclei at ages of 2, 6, 12, and 18 months in *mdx* and *mdx52* mice. Representative immunohistochemical images of maximum clusters of RFs in TA muscles are shown in each group. *Mdx* shows a higher maximum number of RFs than *mdx52* in all age groups. Wild-type C57BL/6 muscle at 2****months old is displayed as a control. An anti-dystrophin C-terminal antibody (green) and DAPI staining (blue) were used. M: months. 20x objective lens, scale bar  = 100****μm.

**Figure 2:**
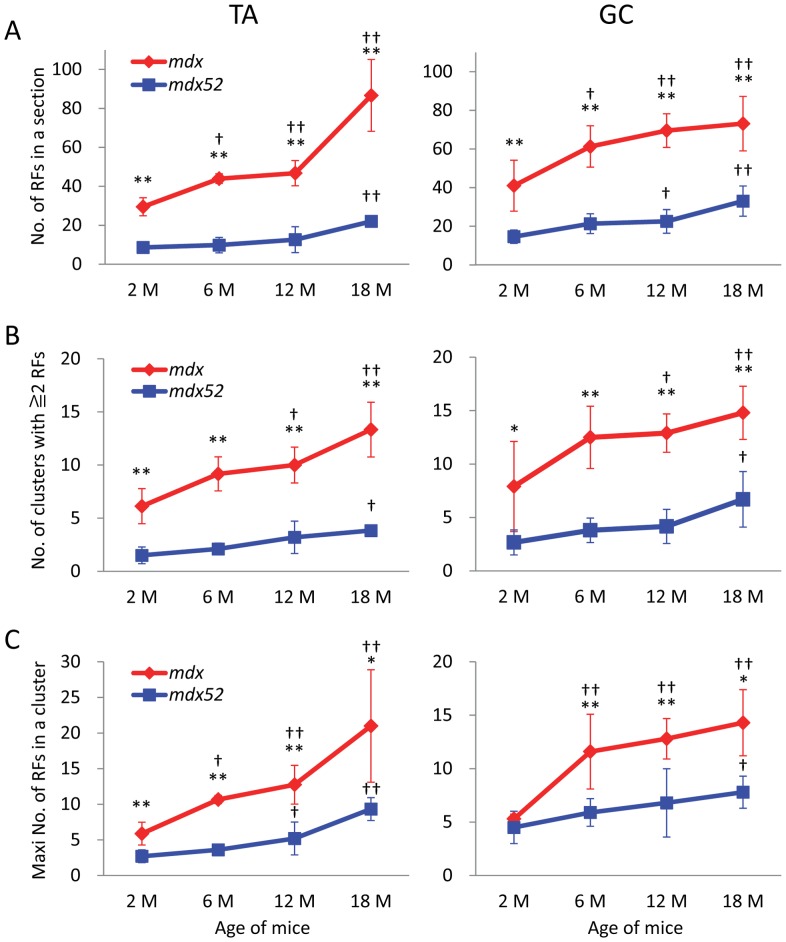
Mutation- and age-related expression of dystrophin-positive revertant fibers in TA and GC muscles from *mdx* and *mdx52* mice. (A) The number of RFs in one TA or GC section. (B) The number of RF clusters containing 2 or more positive fibers. (C) The maximum number of RFs in a single cluster. *Mdx* mice have a significantly higher number of RFs in all criteria than *mdx52* mice except for 2****months of age in maximum number of RFs per cluster. The number of RFs in all criteria increases with age. Values are mean ± S.D. (*n* = 3–6 mice per each group). *P<0.05, **P<0.01 between *mdx* and *mdx52* mice; †*P*<0.05, ††*P*<0.05 compared to 2****months old. M: months.

To investigate the effect of different mutation types of the *DMD* gene on the generation and expansion of RF clusters, we examined RFs in TA and GC muscles of *mdx* mice with C57BL/6 background and *mdx52* mice (also with C57BL/6 background). *Mdx* mice contain a nonsense mutation in exon 23, and *mdx52* mice contain a deletion mutation in exon 52 [36], [37]. RFs were observed in all age groups in both *mdx* and *mdx52* mice and centrally-located nuclei were found in most RFs (**Figure 1**). A rabbit polyclonal antibody against the C-terminal domain of dystrophin was used to detect revertant dystrophin expression because this amino acid-region is reported to be retained in most of the truncated dystrophin or RF proteins in *mdx* and DMD patients [49], [52], [59]. *Mdx* mice clearly showed a higher number of RFs and RF clusters in TA and GC muscles across all age groups in comparison to *mdx52* mice (**Figures 1** **,** **2A, and B**). There were significantly fewer RFs and RF clusters in *mdx52* mice when compared to *mdx* mice. *Mdx* mice also showed a significantly higher number of RFs per cluster compared with *mdx52* mice, except at 2****months of age in GC muscle (**Figure 2C**).

We then compared the expression and clustering levels of RFs at 2, 6, 12, and 18****months of age for *mdx* and *mdx52* strains individually. In TA and GC muscles from *mdx* and *mdx52* mice, the number of RF, RF clusters, and the maximum number of RFs per cluster significantly increased compared to 2****months of age (**Figures 2A, B and C**). The Pearson correlation coefficient showed a strong correlation among these three criteria regarding expression and clustering levels of RFs in each strain (squared correlation coefficient; R^2^ = 0.27–0.84, *P*<0.05). A significant increase in the number of RFs and the maximum number of RFs per cluster at 6****months of age was found only in *mdx* mice. *Mdx* mice at 12****months old also showed significant increase in the number of RF clusters compared to 2****months old. In contrast to the dramatic RF expansion with age in *mdx* mice, *mdx52* mice showed only a moderate increase in RFs with age across all measured criteria. A significant increase in RF expression and clustering was found at 18****months in *mdx* mice compared with *mdx52* mice. Levels of RF expression/clustering in *mdx52* mice never reached levels seen in *mdx* mice. This result is not attributable to genetic background, since both mouse strains have a C57BL/6 background. The *mdx52* mouse, which has fewer RFs and shows little change in RF expansion with age, may be a good model for testing of new therapies aimed at restoring dystrophin expression, because pre-existing RFs can disturb accurate assessments of the restored dystrophin expression owing to therapeutic treatment.

### Dynamics of muscle regeneration with age is different between *mdx* and *mdx52* mice

**Figure 3:**
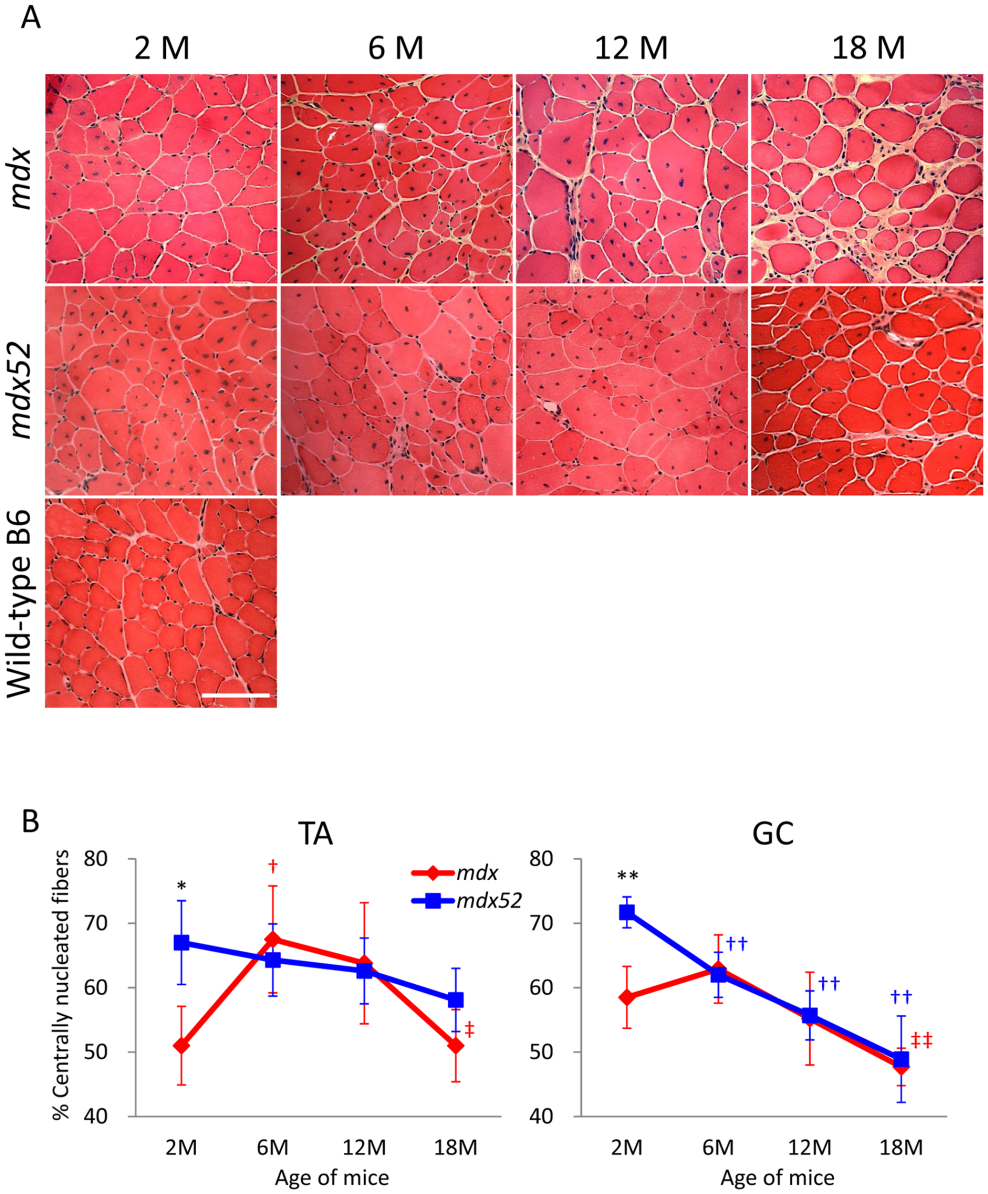
Distinct changes in the percentage of centrally nucleated fibers by mutations and age in *mdx* and *mdx52* mice. (A) Representative images of TA muscles from *mdx* and *mdx52* mice at ages 2, 6, 12 and 18****months with hematoxylin and eosin staining. Wild-type C57BL/6 muscle at 2****months of age is displayed as a control. M: months. Scale bar  = 100****μm. (B) The percentage of centrally nucleated fibers in TA and GC muscles from *mdx* and *mdx52* mice. Three hundred to one thousand myofibers were counted in left and right muscles and the percentage of CNFs was averaged between the two muscles per mouse. Values are mean ± S.D. (*n* = 3–6 mice per group). *P<0.05, **P<0.01 between *mdx* and *mdx52* mice; †*P*<0.05, ††*P*<0.01 compared to 2****months old; ‡*P*<0.05, ‡‡*P*<0.01 compared to 6****months old. Symbol colors are accordant with the color of mice (red; *mdx*, blue; *mdx52*).

To examine whether muscle regeneration is physiologically correlated with RF expansion, we assessed CNFs and eMHC-positive fibers, which are cumulative and current indicators of muscle regeneration, respectively, in *mdx* and *mdx52* mice. During muscle regeneration, regenerated skeletal muscle fibers are centrally nucleated as opposed to mature muscle fibers which are peripherally nucleated [60], [61]. Also, centrally-located nuclei in *mdx* mice are reported to persist long-term, indicating that the percentage of CNFs represents the accumulated history of regeneration up to the present [62], [63]. Following HE staining, *mdx* mice showed a significantly lower percentage of CNFs than *mdx52* mice at 2****months, followed by an increase in percent CNFs resembling observations in *mdx52* mice at 6****months, and a subsequent decrease in CNFs at 12 and 18****months (**Figures 3A and B**). At 18****months old in *mdx* mice, a significant decrease in CNFs was found in both TA and GC muscles when compared to 6****months (the age of the peak CNF percentage). In contrast to the dynamics of CNFs in *mdx* mice, the percentage of CNFs in *mdx52* mice consistently and significantly decreased over time from the peak of 2****months old. This finding indicates that there is a different peak in muscle regeneration between *mdx* and *mdx52* mice, which is before 6****months of age and before 2****months of age, respectively.

**Figure 4:**
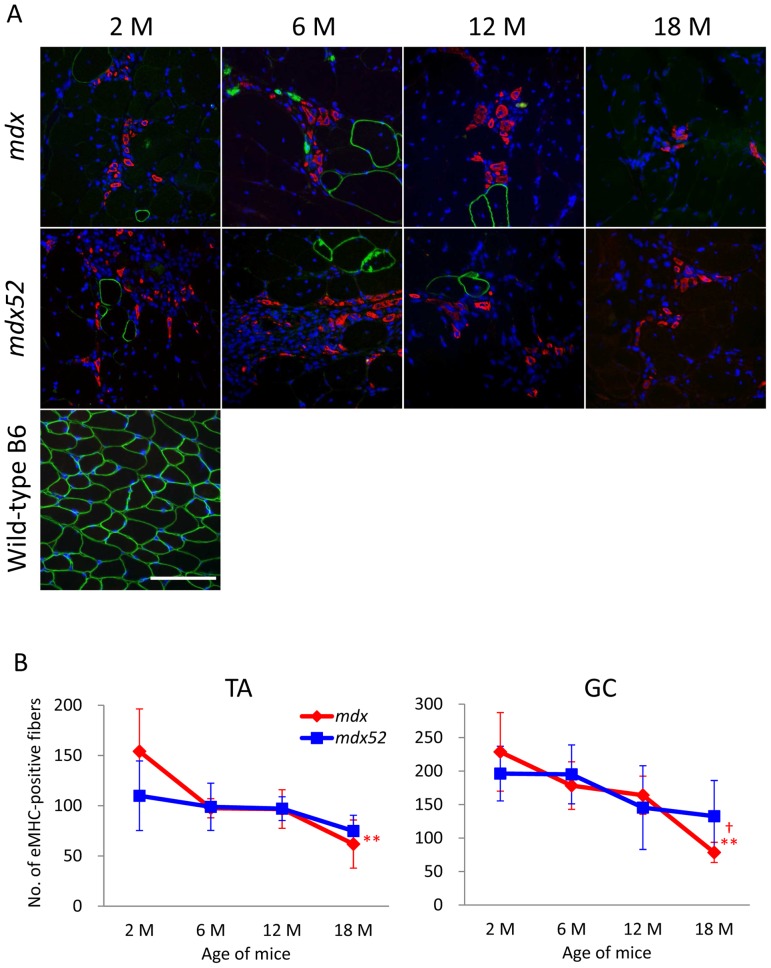
No expression of eMHC in RFs and attenuation of ongoing muscle regeneration in aged *mdx* and *mdx52* mice. (A) Triple staining of *mdx* and *mdx52* mice for RF (green), eMHC (red), and nucleus (blue). Revertant dystrophin is not co-localized with newly regenerated eMHC-positive fibers in TA and GC muscles from *mdx* and *mdx52* mice at any age. The pictures are representative GC muscles from *mdx* and *mdx52* mice at each age. 20x objective lens, scale bar  = 100****μm. (B) The number of eMHC-positive fibers. Values are mean ± S.D. (*n* = 3–6 mice per group). A significant decrease in the number of eMHC-positive fibers is found only at 18****months old in *mdx* mice (**P<0.01 compared to 2****months old, †*P*<0.05 compared to 6 and 12****months old). Symbol colors are accordant with the color of mice (red; *mdx*, blue; *mdx52*).

To examine the possible correlation between ongoing muscle regeneration and RF expansion, we analyzed the co-localization of eMHC with revertant dystrophin and the number of eMHC-positive fibers, which are found short-term during muscle regeneration. Interestingly, eMHC was not expressed in RFs at any age in *mdx* and *mdx52* mice (**Figure 4A**). The number of eMHC-positive fibers was similar between *mdx* and *mdx52* mice at each age (**Figure 4A and B**). However, expression dynamics of eMHC with age was different between the strains. In *mdx* mice, the number of eMHC-positive fibers was the highest at 2****months of age and significantly decreased at 18****months of age in TA and GC muscles, while in *mdx52* mice, the number was slightly but not significantly decreased through 18****months.”. These data from CNFs and eMHC-positive fibers indicate that in *mdx* and *mdx52* mice the dystrophic skeletal muscles actively regenerate throughout their life span but the muscle regeneration activity with age is different between the strains.

## Discussion

Dystrophin RFs were first described more than 20****years ago by Hoffman *et al* [44], however, the mechanisms behind both the generation and expansion of RFs remain poorly understood. Lu *et al* previously reported that revertant events arise in individual muscle satellite cells at around birth [52]. RFs appear at around birth as short segments, approximately 10****µm in length, of sporadic single muscle fibers. One of the most interesting characteristics of the RFs is their substantial expansion with age. In *mdx* mice, RFs continuously expand at least up to 18****months, resulting in clusters containing more than 100 fibers and traversing 1****mm or more of muscle fiber length. However, RFs never accumulate to a sufficient number to significantly ameliorate dystrophic muscle pathology [40].

Our previous study with irradiated *mdx* mice aged up to 2****months supported the hypothesis that muscle regeneration is essential for RF expansion in *mdx* mice [40]. The expansion of RFs has been attributed to the combined effects of cycles of muscle degeneration/regeneration and increased survival of the fibers containing truncated dystrophin [40], [64]. However, in this paper, we demonstrated that mutation types and aging differently affect generation and expansion of dystrophin-positive RFs in *mdx* and *mdx52* mice.

Our paradoxical result reported here strongly challenges our previous hypothesis, since rapid expansion of RFs and a decrease in regenerated CNFs and eMHC-positive fibers occur concurrently at older ages (**Figures 1** **–** **4**). This is in sharp contrast to previous experiments with transgenic *mdx* muscles [40], [65]. No expansion of RFs was observed in any transgenic *mdx* mice expressing mini- or micro-dystrophin in spite of the significant muscle growth before the age of 5 weeks. Instead, the number of RFs decreased with age in these transgenic *mdx* mice. Our observations here lead us to propose two possibilities to explain RF expansion with age: first, mechanisms other than muscle regeneration, such as secondary DNA mutation, up-regulation of short dystrophin isoforms, or age-related changes affecting splicing machinery may be involved in RF expansion in older muscle fibers. The expansion of RFs independent of muscle regeneration supports the existence of a mechanism in aged muscles that Lu *et al* described previously; i.e. RF expansion may represent the progressive increase in a territory of factors, each of which determines a specific pattern of exon skipping (alternative splicing), and which spread by diffusion within each fiber and between neighboring fibers [52]. This hypothesis predicts that RF clusters grow within the existing stable (mature) muscle fibers. A second possibility is that the increase of RFs with age may be related to RF stability combined with the expression of partially functional dystrophin proteins; thus, more stable RFs would accumulate with age and prevent degeneration/regeneration cycles in themselves and in closely surrounding fibers. This could lead to a decrease in muscle regeneration with age in both mouse strains. Nevertheless, muscle regeneration appears to be fundamentally required for RF expansion because central nucleation was found in most RFs through 18****months in this study, and our previous study demonstrated that RF expansion does not occur in the absence of regeneration, even when degeneration continues after irradiation [40]. Another hypothesis to possibly explain these conflicting observations is that regenerating muscle fibers with central nucleation and/or eMHC in older dystrophic mice might change to mature fibers without them more rapidly than in younger dystrophic mice; thus, a reduced number of CNFs and eMHC-positive fibers might not reflect the frequency of regeneration in older muscles, although there is currently no evidence to support this hypothesis.

As no such phenomenon like RFs has been described in other genetic disorders, the mechanism of their genesis and expansion is still open to speculation, although it seems modification of splicing is involved. Thus, RFs could be an interesting model with which to investigate the mechanisms of spontaneous exon skipping (alternative splicing). Future molecular analysis of RFs will also provide invaluable information toward the development of molecular therapies, such as antisense-mediated exon skipping, which are aimed at inducing the production of revertant-like dystrophin-positive fibers for the treatment of DMD [8], [66], [67].
